# The AP2 adaptor enhances clathrin coat stiffness

**DOI:** 10.1111/febs.14961

**Published:** 2019-07-03

**Authors:** Michael Lherbette, Lisa Redlingshöfer, Frances M. Brodsky, Iwan A. T. Schaap, Philip N. Dannhauser

**Affiliations:** ^1^ Institute of Biological Chemistry, Biophysics and Bioengineering, School of Engineering and Physical Sciences Heriot‐Watt University Edinburgh UK; ^2^ Division of Biosciences, Research Department of Structural and Molecular Biology, Institute of Structural and Molecular Biology University College London UK; ^3^ Present address: SmarAct GmbH Oldenburg Germany

**Keywords:** AFM, clathrin, membrane biophysics

## Abstract

Deformation of the plasma membrane into clathrin‐coated vesicles is a critical step in clathrin‐mediated endocytosis and requires the orchestrated assembly of clathrin and endocytic adaptors into a membrane‐associated protein coat. The individual role of these membrane‐bending and curvature‐stabilizing factors is subject to current debate. As such, it is unclear whether the clathrin coat itself is stiff enough to impose curvature and if so, whether this could be effectively transferred to the membrane by the linking adaptor proteins. We have recently demonstrated that clathrin alone is sufficient to form membrane buds *in vitro.* Here, we use atomic force microscopy to assess the contributions of clathrin and its membrane adaptor protein 2 (AP2) to clathrin coat stiffness, which determines the mechanics of vesicle formation. We found that clathrin coats are less than 10‐fold stiffer than the membrane they enclose, suggesting a delicate balance between the forces harnessed from clathrin coat formation and those required for membrane bending. We observed that clathrin adaptor protein AP2 increased the stiffness of coats formed from native clathrin, but did not affect less‐flexible coats formed from clathrin lacking the light chain subunits. We thus propose that clathrin light chains are important for clathrin coat flexibility and that AP2 facilitates efficient cargo sequestration during coated vesicle formation by modulating clathrin coat stiffness.

## Graphical abstract

Formation of clathrin‐coated vesicles facilitates endocytosis of membrane‐embedded cargo proteins. We used atomic force microscopy to establish how specific clathrin coat components contribute to membrane deformation during cargo uptake. We found that the cargo‐binding adaptor protein 2 (AP2) increases coat stiffness, generating a coat better able to deform cargo‐containing membrane. We further found that clathrin light chain subunits provide the clathrin coat with properties that enable AP2 to enhance coat stiffness.

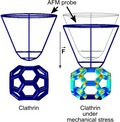

**Abbreviations**
AFM: atomic force microscopy
AP2: adaptor protein 2
CCV: clathrin‐coated vesicle
CHC: clathrin heavy chain
CLC: clathrin light chain
CME: clathrin‐mediated endocytosis

## Introduction

The expression of receptors at the cell surface determines how cells respond and interact with their environment and is thus tightly regulated. Clathrin‐mediated endocytosis (CME) plays an important role in downregulating receptor expression. CME is initiated by adaptor molecules which induce the formation of a polygonal clathrin lattice at the plasma membrane upon recognition of transmembrane cargo including receptors 1. Formation of the clathrin coat promotes local membrane curvature and sequesters cargo into clathrin‐coated vesicles (CCVs) that are excised from the membrane by the GTPase dynamin to remove cargo 1, 2. The assembling clathrin unit is a three‐legged triskelion formed from three clathrin heavy chain (CHC) and three clathrin light chain (CLC) subunits. Clathrin adaptor protein 2 (AP2) is the major adaptor involved in CME, as it is localized to the plasma membrane 3, 4. The mechanics of membrane deformation to generate CCVs have long been a subject of debate. Here, we use atomic force microscopy (AFM) to establish how specific elements of the coat may contribute to the formation of endocytic vesicles at the plasma membrane.

Mathematical modelling of clathrin triskelia and lattice morphology predicted that clathrin coat stiffness would be of similar magnitude as a typical plasma membrane bilayer 5, 6. Thus, it was questioned whether such a flexible clathrin lattice alone could introduce and stabilize membrane curvature 6. Subsequent AFM imaging studies, in which the deformation of isolated CCVs by the imaging probe was measured, predicted that the clathrin coat and membrane layer were only partially coupled by adaptor proteins. It was thus unclear whether membrane deformation can be achieved solely by a stiff protein coat comprising clathrin and accessory proteins, or requires the contribution of membrane‐bending adaptor proteins 7. Using an *in vitro* reconstitution approach, we found that the clathrin lattice alone was sufficient to form vesicles from liposomes. In that system, clathrin assembly was induced on a lipid bilayer by an adaptor fragment that itself is unable to deform membrane but couples the lattice with membrane 2. When liposome rigidity was increased by temperature reduction, the presence of the CLC subunits of clathrin was required to reconstitute this clathrin‐induced vesicle formation 8. From this study, it was not possible to establish the degree to which adaptors might contribute to vesicle formation beyond inducing lattice assembly, and the inferred loose coupling between the clathrin coat and the enclosed vesicle membrane 7 raised the question whether adaptor proteins are sufficiently connected to be involved in transmission of curvature induced by the clathrin coat 9, 10, 11. However, the presence of AP2 restricts the size of a closed clathrin lattice *in vitro*, suggesting that AP2 could influence mechanical properties of a clathrin coat 12 and thereby affect membrane bending.

To shed more light on these issues, we used AFM low force imaging and force spectroscopy to dissect the contribution of individual components to clathrin coat stiffness. Our approach assessed the mechanical properties of clathrin lattices in an aqueous environment by employing the AFM probe for precise nano‐mechanical indentation measurements at spatially well‐defined positions on the coats 13, 14. This enabled direct measurements of the mechanical properties of clathrin coats after manipulating the presence of individual components. In addition, we modelled lattice geometries using finite element methods 14 to better understand the mechanical contributions of the different coat components. Our results uphold the concept of a fine balance between the stiffness of the clathrin coat and the lipid membrane. Incorporation of AP2 into the coat markedly increased the stiffness of the clathrin lattice, suggesting its presence enhances the coat’s capacity to deform membranes.

## Results and Discussion

### Probing clathrin assemblies by AFM

In this study, various clathrin assemblies were investigated (Table 1). In order to get a better understanding of the coupling between the protein coat and the enclosed vesicle membrane, we compared the mechanics of native CCVs comprising clathrin, adaptors and vesicles to the mechanics of detergent‐extracted CCVs (T‐CCVs) lacking enclosed membranes. To dissect the contribution of individual components to clathrin coat performance and thus their influence on CCV formation, we measured the mechanics of *in vitro* assembled clathrin cages from purified, native clathrin or produced from CLC‐free triskelia (CHCs). The effect of adaptors on coat mechanics was assessed by probing structures formed by coassembly of AP2 with native clathrin (AP2 + clathrin) or with CHCs (AP2 + CHC). Clathrin cages to which AP2 was added postassembly (Clathrin + AP2) were also analysed. We will continue to refer to *in vitro* assemblies of clathrin only as ‘cages’ and to assemblies comprising both clathrin and adaptor proteins as ‘coats’.

**Table 1:** Summary of the various clathrin assemblies and the measured stiffness values. AP2, adaptor protein 2; CCV, clathrin‐coated vesicles; CHC, clathrin heavy chain; CLC, clathrin light chain.

Abbreviations	Description	Stiffness
CCVs	Clathrin‐coated vesicles extracted from pig brain: Clathrin coats with enclosed endogenous membrane	0.032 ± 0.009 N·m^−1^ (*n* = 62)
T‐CCVs	TritonX‐100‐treated CCVs: Clathrin coats without internal membranes	0.022 ± 0.006 N·m^−1^ (*n* = 29)
Clathrin (CHC + CLC)	Clathrin cages reconstituted from native purified clathrin (with CHCs and CLCs)	0.024 ± 0.009 N·m^−1^ (*n* = 34)
CHCs	Clathrin cages formed from CHCs (no CLCs)	0.043 ± 0.014 N·m^−1^ (*n* = 53)
CHC cage + CLCs	Clathrin cages formed from CHCs with CLCs added after assembly	0.023 ± 0.006 N·m^−1^ (*n* = 18)
AP2 + clathrin	Clathrin cages reconstituted from native purified clathrin (CHCs + CLCs) coassembled with AP2 adaptor protein	0.044 ± 0.012 N·m^−1^ (*n* = 27)
Clathrin cage + AP2	Clathrin cages reconstituted from native purified clathrin (with CHCs and CLCs) with AP2 adaptor protein added after assembly	0.018 ± 0.004 N·m^−1^, (*n* = 18)
AP2 + CHC	Clathrin cages formed from CHCs coassembled with AP2 adaptor protein	0.050 ± 0.014 N·m^−1^, (*n* = 28)

The mechanics of these various coats and cages were assessed using an AFM force spectroscopy approach that allowed us to measure the local mechanical response at multiple points on a sample as previously described 15. We applied Hooke’s law, which defines the local stiffness of the structure as the ratio between the applied force and the sample indentation, to describe and compare the performance of the different clathrin assemblies and lipid bilayer vesicles. Here, we chose to define the mechanical properties as stiffness rather than the bending stiffness as derived from the thin shell theory. While the latter is often used to describe the mechanics of continuous material such as lipid membranes 16, this model may be less applicable to clathrin lattices where clathrin triskelia form a fixed network rather than a continuous material 17.

**Figure 1:**
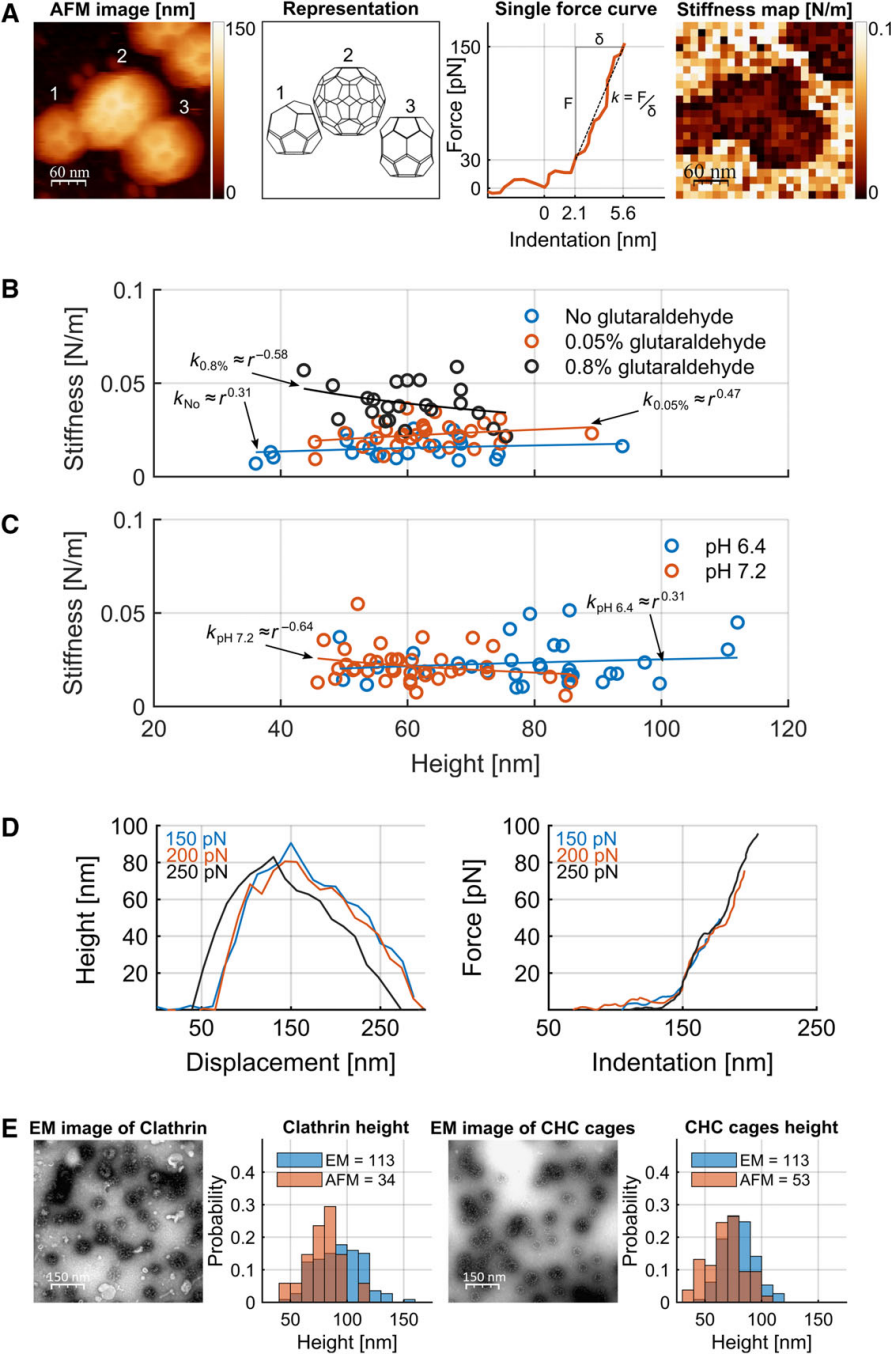
Force measurements on clathrin assemblies by AFM. (A) AFM topography scan (left) and stiffness map (right) covering three individual clathrin cages. Brighter colours indicate higher stiffness. The three clathrin cages have distinct architecture (middle left): a mini coat of about 60 nm height (1), a truncated icosahedron of 80 nm height (2), and a 70 nm hexagonal barrel (3). A typical single force curve shows exerted force in relation to the indentation of the cage (middle right). One force curve is obtained per pixel of the stiffness map (right). The indentation region, defined as the region of forces, F, between 30 and 150 pN, and indentations, δ, between 2.1 and 5.6 nm, is linearly fitted to obtain the stiffness, *k*, of the cage (dotted black line). The contact point at 30 pN defines the height of the structure. The overall stiffness of a particular clathrin assembly is determined from the average of all force curves within 20 nm radius of the apex of the structure. *Z* scale to the right: dark brown to white represents a range of height from 0 to 150 nm. (B) The effect of glutaraldehyde on stiffness of T‐CCVs measured by AFM. Stiffness of coats fixed with 0% (blue), 0.05% (red) and 0.8% (black) glutaraldehyde. T‐CCV stiffness was plotted in relation to size, and data fitted according to a spherical shell model (see Methods). The average stiffness was 0.017 ± 0.004 N·m^−1^ (*n* = 23), 0.022 ± 0.006 N·m^−1^ (*n* = 29) and 0.033 ± 0.008 N·m^−1^ (*n* = 22), at 0%, 0.5% and 0.8% glutaraldehyde respectively. (C) Effect of pH on clathrin cage stiffness treated with 0.05% of glutaraldehyde. The average stiffness of cages at pH 6.4 (blue) was 0.024 ± 0.009 N·m^−1^ (*n* = 34) and at pH 7.2 (red) was 0.018 ± 0.006 N·m^−1^ (*n* = 39). No statistical difference was found between the two populations (*t*‐test, *P < 0.05, *P* = 0.53 assuming equal variance). The values for *k* were obtained by fitting the data with a power law function (solid lines, see Methods). (D) Left: Cross‐sectional height topographs produced from three successive mechanical measurements with increasing force on one cage. Right: Force curves corresponding to the same measurements of increasing maximum indentation force: 150 pN (blue), 200 pN (red) and 250 pN (black)*.* (E) Electron micrographs and size histograms of clathrin or CHC cages as determined by AFM (as in A) or by EM. Mean size of clathrin cages was 78.4 ± 2.7 nm from AFM (mean ± SEM; *n* = 34, red) and 92.6 ± 2.0 nm from EM (mean ± SEM; *n* = 113, blue) and for CHC cages was 67.9 ± 2.1 nm from AFM (mean ± SEM; *n* = 53, red) and 79.3 ± 1.4 nm from EM (mean ± SEM; *n* = 113, blue). The probability gives the normalized count to allow a direct comparison between histograms.

The integrity of each structure was first confirmed by AFM amplitude modulation imaging using soft cantilevers oscillating with an amplitude of approximately 7 nm. Under these conditions, the exerted force was in the range of tens of pN, which represents the lowest force limit of AFM 18. Our method allowed us to clearly distinguish different cage structures such as the truncated triakis tetrahedron made of 12 pentagons and 4 hexagons, the hexagonal barrel comprising 12 pentagons and 8 hexagons, and the truncated icosahedron that consists of 12 pentagons and 20 hexagons (Fig. 1A). Then, a 2D array of force spectroscopy curves, also known as force mapping, over the whole structure was performed. From each force spectroscopy curve, we determined the contact point (the tip position where the applied force exceeds the AFM noise threshold 18) and the slope of the indentation region to reconstruct height and stiffness maps of the sample, respectively (Fig. 1A). The latter thereby spatially describes the mechanical properties of the structure.

To stabilize samples for prolonged AFM measurements, all samples were treated with 0.05% glutaraldehyde, which increased the stiffness of clathrin cages by approximately 30% compared to unfixed cages (Fig. 1B). The stiffness of these stabilized clathrin cages were similar under our working conditions (pH 6.4) and at physiological pH (Fig. 1C). Experimental conditions were kept identical to allow for comparative measurements between samples.

Next, we assessed whether the elastic properties and the height of each structure were preserved after the generation of multiple force maps on the same individual clathrin cages (Fig. 1D). No significant changes in cage height and stiffness were detected after three successive force measurements with increasing maximum force (150, 200 and 250 pN). This consistency confirmed that the cage was not irreversibly deformed. Furthermore, the averaged force curves at the apex of the cage showed identical slopes in the indentation region, thus identical cage stiffness, demonstrating that the forces applied were within the elastic range of the sample. To further validate that the structures withstand the AFM imaging procedure without major deformation, we compared the heights of clathrin and CHC cages measured by AFM to the diameters obtained from standard electron microscopy (EM) images of negatively stained samples 19, 20. We found that for both cage types the heights measured by AFM were on average about 15% smaller than the diameters measured by EM (Fig. 1E), a magnitude of discrepancy that had been observed in earlier AFM studies 15. EM may yield slightly larger measurements due to the presence of uranyl acetate used to visualize clathrin structures.

### The native clathrin coat is flexible and loosely coupled with the enclosed lipid vesicle

**Figure 2:**
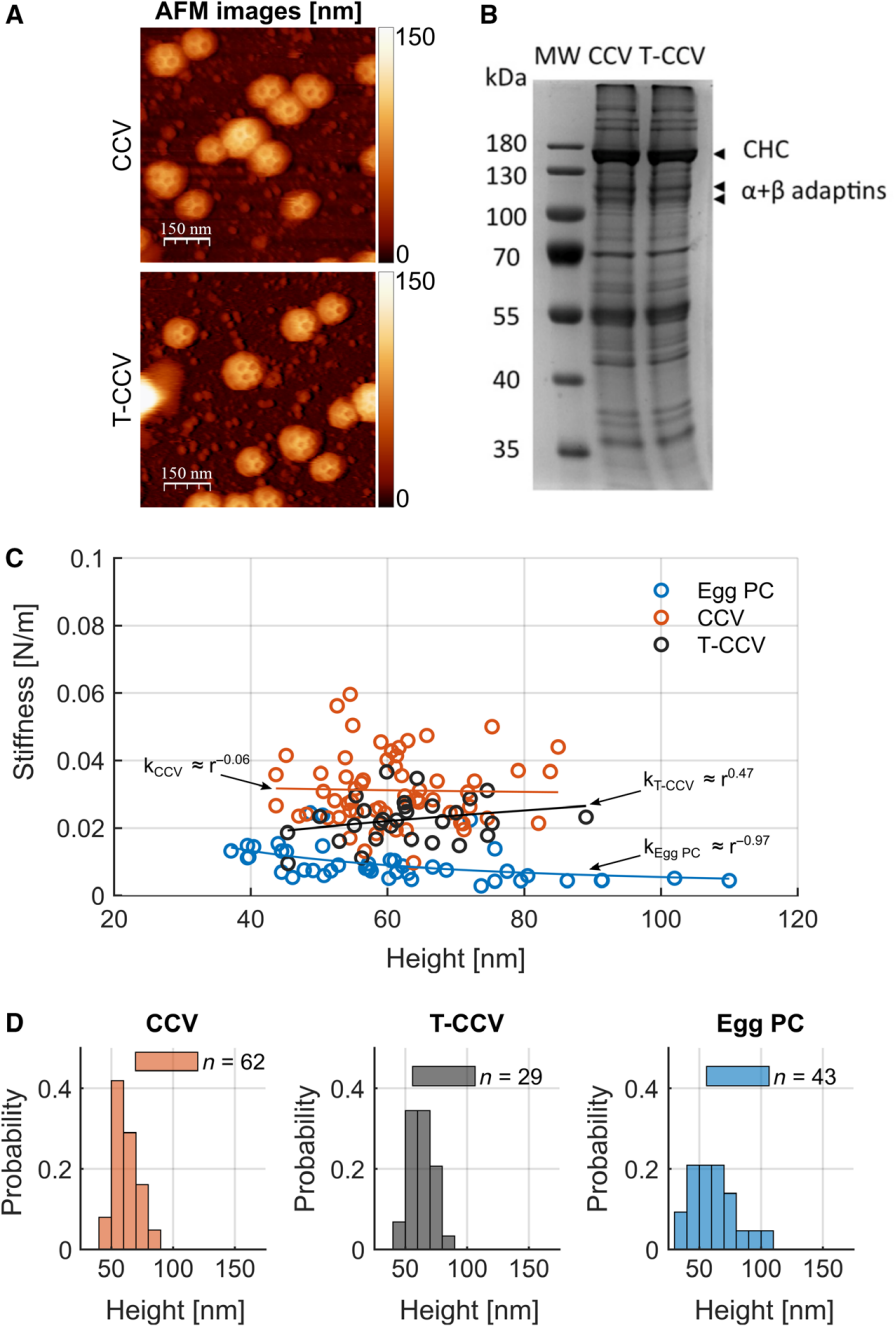
Mechanical properties of CCVs and their individual layer components. (A) AFM topographs of CCVs and T‐CCVs. *Z* scale to the right: dark brown to white represents a range of height from 0 to 150 nm. (B) Coomassie‐stained SDS/PAGE showing the protein profiles of CCVs and TritonX‐100–treated CCVs (T‐CCVs). The masses (kilodaltons, kDa) of the molecular weight (MW) marker proteins in the indicated lane are listed to the left of the migration position of each protein. The migration positions of CHC and the alpha and beta subunits of the AP2 adaptor (α + β adaptins) are indicated at the right. (C) The stiffness of native CCVs in comparison to T‐CCVs and model membrane vesicles (produced from egg phosphocholine, egg PC) measured by AFM. The stiffness of the CCVs is 0.032 ± 0.009 N·m^−1^ (*n* = 62, red) and does not show a clear correlation with size. For comparison, the stiffness of PC vesicles was determined to be 0.007 ± 0.004 N·m^−1^ (*n* = 43, blue, data reproduced from Schaap *et al*. 15). The T‐CCV stiffness is lower compared to intact CCVs with 0.022 ± 0.006 N·m^−1^ (*n* = 29, black). The values for *k* were obtained by fitting the data with a power law function (solid lines, see Methods). (D) Size histograms of CCV, T‐CCV and egg PC vesicle samples as in (C) determined by AFM in c. Mean size of CCVs was 61.1 ± 1.2 nm (mean ± SEM; *n* = 62, red), of T‐CCVs was 62.7 ± 1.8 nm (mean ± SEM; *n* = 27, black) and of egg PC vesicles was 61.3 ± 2.7 nm (mean ± SEM; *n* = 43, blue). The probability gives the normalized count to allow a direct comparison between histograms.

The mechanics of intact CCVs was previously evaluated from the induced deformation by AFM imaging of the coats. With these imaging‐based measurements, the strength of the coupling between the coat and lipid layer was estimated by comparing the measured coat response with reported values for the membrane‐bending rigidity 7. We revisited this issue using AFM force spectroscopy rather than AFM imaging, which allowed for a direct measurement of the structural performance of the coats, cages and lipid bilayer vesicles. This approach made it possible to compare the stiffness of native CCVs with that of CCVs treated with detergent to dissolve the internal membrane vesicle (T‐CCVs). Well‐defined polyhedral clathrin lattices were observed for both structures, with size ranges of 40–90 nm for each (Fig. 2A,D). Analysis of the protein composition of CCVs and T‐CCVs by SDS/PAGE and Coomassie staining showed no major changes in protein composition with respect to CHCs and adaptor proteins (Fig. 2B), indicating that clathrin coat integrity was preserved during detergent treatment, as previously described 21. Smaller structures were slightly stiffer than larger ones (Fig. 2C), a trend that would be expected for thin spherical shells for which stiffness scales with the reciprocal of the radius (*r* ^−1^) 22. To account for this when comparing different samples, data were fitted to a *r* ^α^ function in order to obtain the stiffness for an 80 nm high object and errors were calculated as mean absolute error (MAE).

We thereby calculated the stiffness of CCVs as 0.032 ± 0.009 N·m^−1^, *n* = 62, (Fig. 2C), about fourfold greater than the reported stiffness of phosphatidylcholine lipid vesicles derived from egg (egg PC) of comparable size (0.007 ± 0.004 N·m^−1^, *n* = 43 as determined by Schaap *et al*. 15). T‐CCVs were slightly less stiff than CCVs (0.022 ± 0.006 N·m^−1^, *n* = 29, Fig. 2C), indicating that the stiffness of an intact CCV is approximately the sum rather than the product of the protein coat and the enclosed vesicle stiffness. Thus, the two layers of a CCV can be considered a mechanical system of two springs in parallel rather than in series. This is consistent with the notion that these two layers are only loosely coupled via their adaptor proteins. Strong coupling between the two layers would generate up to 100‐fold higher stiffness of the CCV than the clathrin coat (T‐CCV) or membrane vesicle alone 7. The observed loose coupling might result from the relative flexible nature of the adaptor proteins' link to clathrin 23, as well as from limited physical connection of the clathrin coat to the lipid vesicle by a substoichiometric ratio of adaptors to triskelia 24, 25. CCVs are about one order of magnitude less stiff than capsids from viruses like adenovirus 26 or Herpes virus 27, which are protein assemblies of similar dimensions and have a stiffness in the range of ~ 0.4 N·m^−1^. For viruses the stiffness is defined mainly by the protein capsid, whereas the enclosed genome only has a small effect 28. A comparatively flexible clathrin coat may have been selected for during evolution to facilitate rapid vesicle formation and uncoating, while viral protein capsids enclosing the viral genomes will have evolved towards maximum stability in order to provide structural support and protection of the RNA or DNA molecules.

### Contributions of individual components to clathrin coat stiffness

**Figure 3:**
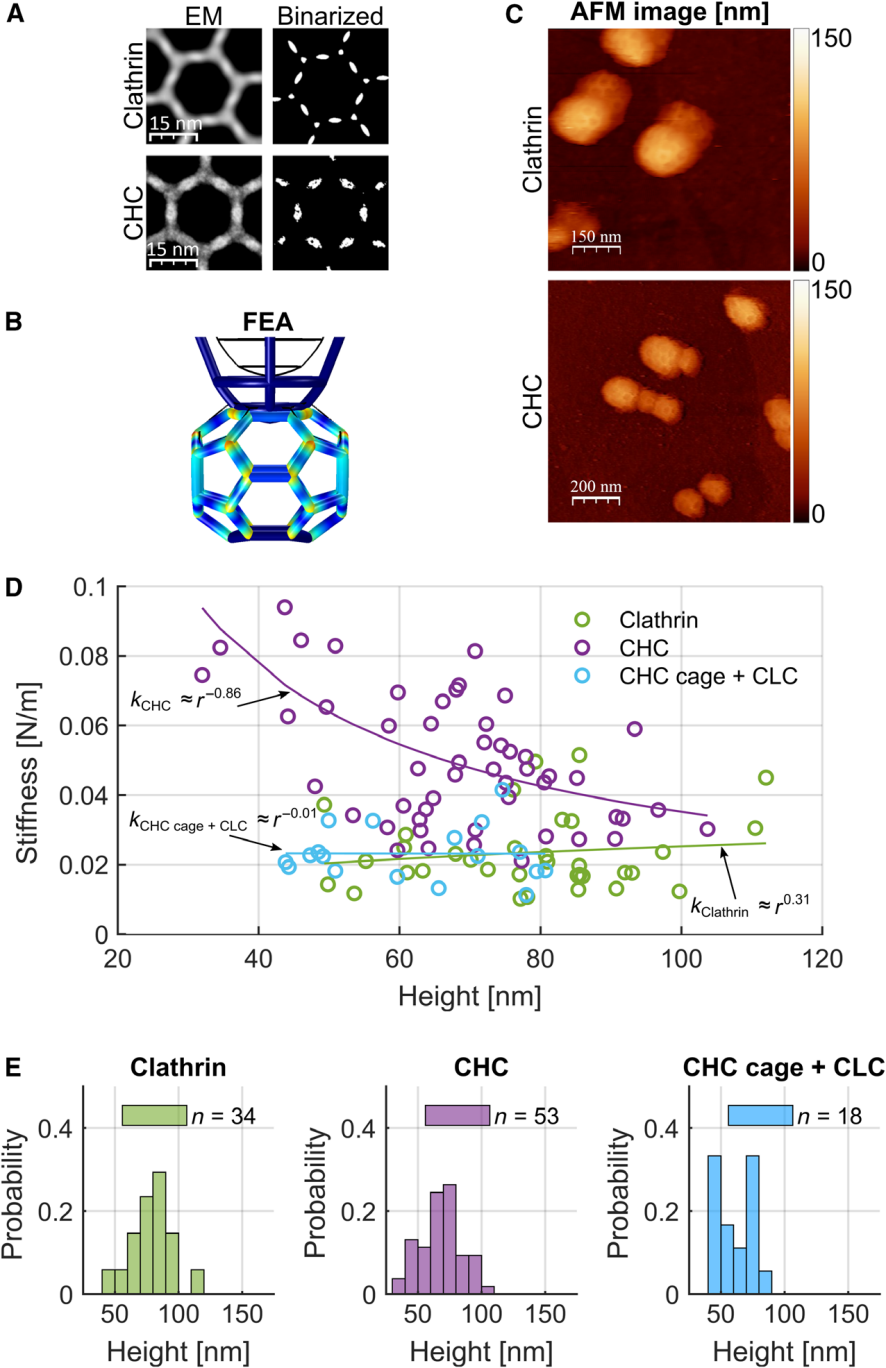
Effect of CLCs on the structure and mechanics of clathrin coats. (A) Left: Average pictures of 2D clathrin and CHC lattices, negatively stained with uranyl acetate and imaged by EM. Right: Average pictures after binary transformation to enhance the visualization of the distribution of image intensity as a measure for the protein density. (B) FEA simulation of a hexagonal barrel being indented by a parabolic tip. The stress within the structure is visualized by a colour gradient from blue (lowest) through yellow to red (highest) stress. (C) AFM topographs of clathrin and CHC cages. A double tip effect is visible in the topograph of clathrin. *Z* scale to the right: dark brown to white represents a range of height from 0 to 150 nm. (D) Stiffness of clathrin cages, CHC cages and CHC cages to which CLCs were added after assembly (CHC cage + CLC) measured by AFM. Stiffness of CHC cages: 0.043 ± 0.014 N·m^−1^ (*n* = 53, purple); clathrin cages: 0.024 ± 0.009 N·m^−1^ (*n* = 34, green); CHC cages + CLC: 0.023 ± 0.006 N·m^−1^ (*n* = 18, light blue). The values for *k* were obtained by fitting the data with a power law function (solid lines, see Methods). (E) Size histograms of clathrin, CHC and CHC cage + CLC cages as in (D) determined by AFM. Mean size of clathrin cages was 78.4 ± 2.7 nm (mean ± SEM; *n* = 34, green), of CHC cages was 67.9 ± 2.1 nm (mean ± SEM; *n* = 53, purple) and of CHC cages + CLC was 62.0 ± 3.1 nm (mean ± SEM; *n* = 18, blue). The probability gives the normalized count to allow a direct comparison between histograms.

To dissect the contribution of individual components to clathrin coat stiffness, we next investigated how clathrin coat stiffness is influenced by its most abundant components – the two subunits of clathrin itself and AP2. We used image averaging to compare hexagons from electron micrographs of planar clathrin lattices formed from native clathrin and CHCs. Protein densities differed near the lattice nodes, where the CLCs bind the CHC trimerization domain, while the overall hexagon structures were similar. This indicates that mainly the triskelion pucker conformation at the lattice nodes is altered in the absence of CLCs (Fig. 3A). In line with this, CHC triskelia were previously observed to lose their natural pucker within planar clathrin assemblies when compared to assemblies of native clathrin, demonstrating that CLCs stabilize the puckered conformation of clathrin triskelia 8, 29.

To predict whether lattice node stability is important for the stiffness of a clathrin cage, we simulated the compression of a clathrin cage, modelled as a hexagonal barrel, by an AFM probe using finite element analysis (FEA). This revealed that the highest structural stress was concentrated in the nodes (indicated by warm colours, Fig. 3B). Taken together, this led us to hypothesize that through their interaction with the trimerisation domain, CLCs possibly influence the stiffness of a clathrin cage 29. To test this, we compared the stiffness of cages assembled from native clathrin and CHCs *in vitro* (Fig. 3C) and found that CHC cages were about two times stiffer than native clathrin cages (0.043 ± 0.014 N·m^−1^, *n* = 53, compared to 0.024 ± 0.009 N·m^−1^, *n* = 34, Fig. 3D). When CLCs were added to preassembled CHC cages (CHC cages + CLCs), the stiffness was reduced to a level similar to native clathrin cages (0.023 ± 0.006 N·m^−1^; *n* = 18, Fig. 3D), confirming that CLCs influence the mechanical properties of clathrin cages. The comparison of cage size distributions of clathrin, CHC and CHC + CLC cages reveals that clathrin cages have larger diameters. In conjunction with previous reports 20, cages assembled without CLCs are smaller, even when CLCs were added afterwards (Fig. 3E). One possible explanation for this difference could be the influence of the CLCs on the triskelion pucker during clathrin cage assembly. In particular, it appears that CLCs increase lattice flexibility while simultaneously maintaining mechanical stability, rather than simply enhancing rigidity as previously assumed 8. The latter assumption was based on the requirement for CLCs to deform phospholipid vesicles at 15 °C, while they are not required to sustain budding at 37 °C 8. As CLCs bind to CHC with high affinity and have a very low exchange rate 30, 31, regulation of coat properties by transient CLC binding is unlikely. CLCs are dispensable for endocytosis of many, but not all cargo molecules 32. In the light of the observed differences in mechanical properties between native and CLC‐free clathrin, it may be that the CLC influence on lattice flexibility enables uptake of certain cargo that alter membrane‐bending properties.

**Figure 4:**
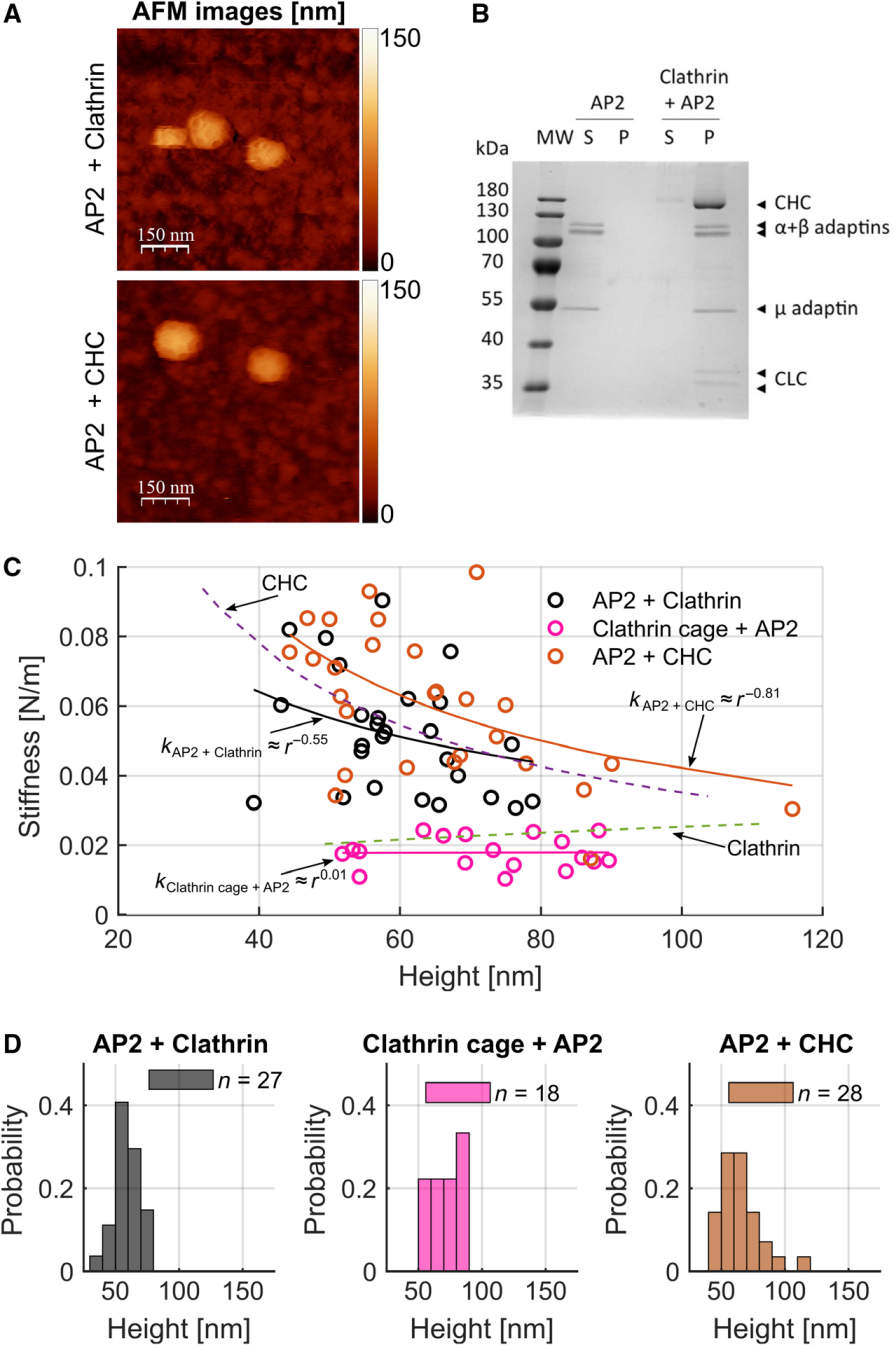
Effect of adaptor protein AP2 on clathrin coat rigidity. (A) AFM topographic images of AP2 + clathrin and AP2 + CHC cages. *Z* scale to the right: dark brown to white represents a range of height from 0 to 150 nm. (B) AP2 was dialysed overnight either alone or together with clathrin triskelia into buffer A, in a ratio of 3 : 1 (w/w) (CHC : AP2). Assemblies were then pelleted by ultracentrifugation and protein composition analysed by SDS/PAGE, Coomassie staining and densitometry. The masses (kDa) of the MW marker proteins in the indicated lane are listed to the left of the migration position of each protein. The migration positions of CHC, the two vertebrate CLC and the alpha, beta and mu subunits of the AP2 adaptor (α + β, µ adaptins) are indicated at the right. S, Supernatant, P, Pellet. (C) Stiffness of clathrin and CHC coassemblies with AP2 and clathrin cages with AP2 added after assembly measured by AFM. Average stiffnesses were AP2 + clathrin cages (0.044 ± 0.012 N·m^−1^ *n* = 27, black), clathrin cages to which AP2 was added (clathrin cages + AP2, 0.018 ± 0.004 N·m^−1^, *n* = 18, pink) and AP2 + CHC cages (0.050 ± 0.014 N·m^−1^, *n* = 28, brown). The fits of the clathrin cages (green, dotted line) and CHCs (purple, dotted line) as shown in Fig. 3D are displayed for comparison. The values for *k* were obtained by fitting the data with a power law function (solid lines, see Methods). (D) Size histograms of AP2 + clathrin, clathrin cages + AP2 and AP2 + CHC cages as in C) determined by AFM. The AP2 + clathrin cages were 1.3 times smaller than clathrin cages with an average height of 59.7 ± 2.0 nm (mean ± SEM; *n* = 27, black) while heights of clathrin cages + AP2 were 72.4 ± 3.1 nm (mean ± SEM; *n* = 18, pink) and AP2 + CHC cages were 64.7 ± 3.0 nm (mean ± SEM; *n* = 28, brown). The probability gives the normalized count to allow a direct comparison between histograms.

Next, we tested the influence of adaptor proteins on clathrin coat stiffness by assessing the stiffness of clathrin coats assembled *in vitro* from mixtures of native clathrin or CLC‐free clathrin (CHC) with AP2 (Fig. 4A). Incorporation of AP2 into coats was confirmed by sedimentation analysis (Fig. 4B). Consistent with previous reports 12, 33, clathrin coats were found to be about 1.3 times smaller than clathrin cages formed before adding AP2 or lacking AP2 (Fig. 4D). Coassembly of CHCs with AP2 did not lead to a further decrease in size 34 (Figs 4D and 3E). The stiffness of clathrin coats (0.044 ± 0.012 N·m^−1^, *n* = 27) was markedly increased compared to the stiffness of clathrin cages lacking AP2 (0.024 ± 0.009 N·m^−1^, *n* = 34, Fig. 4C) and was similar to that of CHC cages. The addition of AP2 to preassembled clathrin cages did not influence their stiffness (0.018 ± 0.004 N·m^−1^, *n* = 18, Fig. 4C). Neither did the incorporation of AP2 into CLC‐free clathrin assemblies lead to a further increase in stiffness (0.050 ± 0.014 N·m^−1^; *n* = 28, Fig. 4C). Thus, it appears that CLC‐free clathrin assemblies are too inflexible to be influenced by AP2. Conversely, clathrin cages are more flexible if CLCs are bound, and lattice stiffness can be increased by incorporation of AP2 into the assembling coat. Inflexibility could explain why CLC‐free clathrin is unable to deform membrane at low temperatures, as CLC‐free lattices may be less able to introduce curvature during membrane invagination *in vitro*. In living cells, CLCs are required for the uptake of some, but not all clathrin‐dependent cargo 32. Furthermore, CLCs prevent spontaneous clathrin assembly at cellular pH 20, 35, rendering assembly dependent upon initiation by adaptor proteins such as AP2 since CLCs do not significantly dissociate from heavy chain subunits of clathrin under physiological conditions 30, 31. Here, we show that clathrin cages are in fact stiffer and less susceptible to regulation by AP2 in the absence of CLCs. Thus, it appears that CLCs influence clathrin mechanics, but differently than previously hypothesized, and that AP2 plays a critical role in regulating coat stiffness. Our data suggest that the presence of CLCs is required for AP2 to influence coat stiffness, which could affect uptake of cargoes that increase membrane rigidity.

While clathrin assembly alone is sufficient to facilitate membrane deformation *in vitro* 2, it is less clear whether clathrin coat stiffness is sufficient to induce and stabilize membrane curvature within cells. We found that clathrin assemblies were stiffer than lipid membrane, yet within the same order of magnitude. We hypothesize that this fine balance could serve as a point of regulation of endocytosis. The increase in coat stiffness by incorporation of AP2 during clathrin assembly may provide a mechanism to couple efficient cargo sequestration with vesicle formation. In line with this, AP2 has been demonstrated to be crucial for stabilizing nascent clathrin‐coated pits and enhances CCV maturation efficiency through cargo concentration 36. Furthermore, it has recently been shown that the ratio between AP2 and clathrin changes over the time course of CCV formation, where the transition from a flat into a curved clathrin lattice occurs at the point when incorporation of additional AP2 into the coat reaches a plateau 37. In conjunction with this observation, our findings would suggest that AP2 incorporation increases coat stiffness to a level that exceeds the stiffness of the membrane in order to support the initiation of membrane curvature. In addition, AP2‐mediated rigidification of the clathrin coat could also serve to counteract increased resistance of membrane deformation from accumulating cargo recruited by AP2. Crowding of cargo molecules within a growing clathrin‐coated pit can create steric pressure 38 and increase the energy barrier to deform membrane requiring increased coat stiffness 39 for vesicle formation. The mechanism we propose seems to be particularly important in the initial phase of CCV formation. Once initial curvature has been generated and stabilized, the clathrin coat may be able to grow along the edges without further incorporation of AP2, resulting in declining AP2/clathrin rations during vesicle maturation 37 and would explain the overall loose coupling of the protein shell to the enclosed vesicle membrane in the final CCV.

## Methods

### Protein purification

Clathrin‐coated vesicles were purified from porcine brain tissue as described previously 2, 19, 40.

The T‐CCVs were prepared by incubating CCVs in 1% Triton in buffer A [100 mm 2‐(N‐morpholino)‐ethanesulfonic acid, 1 mm EDTA, 0.5 mm MgCl_2_ and 2 mm CaCl_2_, 0.02% NaN_3_, pH 6.4] for 2 h on ice. T‐CCVs were then collected by centrifugation (109 000 ***g*** for 30 min at 4 °C) and resuspended in buffer A.

Clathrin triskelia and AP2 were purified from porcine brain tissue by size exclusion (Superose 6; GE Healthcare Life Sciences, Freiburg, Germany) and ion‐exchange (hydroxyapatite; BioRad, Basel, Switzerland) chromatography as described elsewhere 41, 42, 43. Light chain‐free CHCs were purified as described previously 20, 31.

Alternatively, clathrin and CHC were purified by gel/affinity chromatography over CaptoCore 700 columns (GE Healthcare Life Sciences). To this end, 1 mL of CCVs were pelleted at 109 000 ***g*** for 30 min at 4 °C. The pellet was then resuspended in 1 mL 10 mm Hepes pH 8.5, homogenized using a SS30 dounce homogenizer (Stuart, Staffordshire, UK) and incubated for 10 min on ice. Membranes and most of the remaining proteins were then removed by two successive centrifugation steps at 149 000 ***g*** for 30 min at 4 °C. The supernatant was run through a 1 mL CaptoCore700 column (GE Healthcare Life Sciences) either automated at 0.5 mL·min^−1^ or by gravity flow. 0.5 mL fractions were collected and analysed by SDS/PAGE and Coomassie/Immunoblotting. Pure, CHC‐rich fractions were pooled and concentrated by centrifugation in centricon 100 kDa (GE Healthcare Life Sciences). Concentrated clathrin/CHC was dialysed first against 0.5 m Tris for 2 h, then against buffer A + 2 mm calcium overnight.

Clathrin was stored as reassembled cages (see below) in buffer A at 4 °C and AP2 in 0.5 m Tris, 20% glycerol pH 7.4 at −80 °C.

### Clathrin cage and coat assembly

For assembly of clathrin cages, native clathrin or CLC‐free CHC was dialysed overnight into buffer A at concentrations between 0.5 and 1 mg·mL^−1^. For coat assembly, triskelia and AP2 were mixed in a ratio of 3 : 1 (w/w) and dialysed in buffer A overnight 33. Binding of AP2 to preassembled cages was facilitated by mixing cages with AP2 in a ratio of 3 : 1 (w/w) in buffer A and incubated for 1 h on ice. Cages were recovered by centrifugation (109 000 ***g*** for 30 min at 4 °C) and resuspended in buffer A. Adaptor binding was confirmed by SDS/PAGE analysis.

### EM sample preparation

For negative staining of CCVs and clathrin or CHC cages, freshly glow‐discharged carbon‐coated formvar grids were used. Typically, sample volumes between 5 and 10 µL were applied to the grids for 90 s. After rinsing the grids twice with buffer A, samples were stained for 1 min with 2% aqueous uranyl acetate. Specimens were imaged with a Tecnai spirit (FEI) transmission electron microscope at an acceleration voltage of 120 kV. Electron micrographs were processed using imagej (NIH, Bethesda, MD, USA).

### AFM sample preparation

We used Highly Oriented Pyrolytic Graphite (HOPG; Micromasch) as substrate for the AFM experiments. The surface was plasma cleaned for 135 s and 20 µL of 0.02 m cages was allowed to adsorb for 1 min. To remove nonadsorbed cages, the surface was washed twice with 100 µL of buffer A. Then, 0.05% of glutaraldehyde was added for 10 min after which the surface was washed again with buffer A to remove any unbound molecules.

### Atomic force microscopy

All experiments were performed on an MFP‐3D AFM (Asylum Research) at room temperature. We used RC150VB cantilevers (Olympus, Hamburg, Germany) with a resonant frequency in liquid of approximately 4 kHz. The spring constant was calibrated for each cantilever with the built‐in calibration routine based on the thermal noise method (0.031 ± 0.001 N·m^−1^, mean ± SEM, *n* = 42).

All pictures were obtained in amplitude modulation mode (scan rate of 1 Hz, amplitude of ~ 7 nm), and all force maps were performed over an area of 300 × 300 nm and recording a total of 24 × 24 force curves per map. Each force curve was performed by a 500 nm displacement of the z‐scanner with a speed of 2 µm·s^−1^. The curves were converted from force *vs*. distance into force *vs*. indentation curves using established procedures 14.

### Determination of cage size by AFM and EM

The height of the cages and coats imaged by AFM were determined based on the conversion of the recorded force maps into height maps. The heights of a structure were defined as the difference between the contact point of an average of nine background curves and the contact point of individual force curves on top of the structure. The contact point is defined as the position of the tip at which the force exceeded 30 pN. The sizes of the coats imaged by EM were determined by measuring the diameter of negatively stained cages using imagej.

### Force curve analysis

Force curves were analysed using a modified version of a previously described analysis routine 15. Briefly, force curves within 20 nm radius to the apex of the individual cage or coat were interpolated and aligned before averaging. The indentation region between 30 and 150 pN was linearly fitted to extract its slope, corresponding to the stiffness (in N·m^−1^) of the structure according to Hooke’s law.

To compare stiffness values between sample types, data were fitted using a power law function written as *k *(*r*) = *a* ·*r* ^α^, where *r* is the radius of the cage, *a* being a scaling prefactor and α, a dimensionless coefficient that describes the correlation between the height and the stiffness. Coefficients were obtained by iterative least squares estimation. Then, the fitted stiffness value of a structure of 80 nm in diameter, a typical size for clathrin assemblies 2, was chosen to compare the coats of the different samples. Errors due to the regression were estimated by MAE of the residuals.

### Finite element analysis

We modelled the coats and cages as beam structures with circular cross section that have rigid nodes and a Young’s modulus of 100 MPa using Comsol 5.2a (Comsol). To simulate AFM experiments, the edges of the lattice hexagon or pentagon in contact with the substrate were constrained in all directions. We used the contact‐penalty method to implement the contact between the spherical apex of the AFM tip (radius 20 nm) and the top of the cage 14. The tip was positioned few nanometres above the highest point of the structure and then lowered stepwise. The simulation was stopped when the force exerted onto the structure exceeded 150 pN.

### Averaging of EM clathrin lattices

The sample preparation and image reconstruction of planar lattices has been described in earlier work 8. Briefly, electron micrographs of two‐dimensional clathrin lattices were cropped in multiple subfigures of 100 × 100 nm (97 for clathrin and 73 for CHC), each containing a single hexagon in their centre and surrounded by six adjacent hexagons. The number of images was multiplied by six by their rotation in steps of 60° to make use of their sixfold symmetry. Next, all images were aligned by maximizing the cross correlation and an average image was obtained. All images where then again aligned with respect to the averaged image. This procedure was repeated in an iterative fashion until their cross correlation did not further increase.

## Conflict of interest

The authors declare no conflict of interest.

## Author contributions

LR and ML contributed equally to this work. AFM measurements were performed by ML. FEA simulation and EM image averaging was performed by ML and IATS. Protein samples were produced by LR and PND. EM imaging was performed by LR. Experiments were conceived by LR, PND, ML and IATS. The manuscript was written by LR, ML, IATS, FMB and PND.
